# *Lactobacillus reuteri* Ameliorates Lipopolysaccharide-Induced Acute Lung Injury by Modulating the Gut Microbiota in Mice

**DOI:** 10.3390/nu15194256

**Published:** 2023-10-04

**Authors:** Jian Shen, Shuting Wang, Yong Huang, Zhengjie Wu, Shengyi Han, He Xia, Hui Chen, Lanjuan Li

**Affiliations:** 1 State Key Laboratory for Diagnosis and Treatment of Infectious Diseases, National Clinical Research Centre for Infectious Diseases, Collaborative Innovation Centre for Diagnosis and Treatment of Infectious Diseases, The First Affiliated Hospital, Zhejiang University School of Medicine, 79 Qingchun Rd., Hangzhou 310003, China; 2 Department of Gastroenterology, The Second Affiliated Hospital of Nanchang University, Nanchang 330006, China; 3 Department of Infectious Disease, Shulan (Hangzhou) Hospital Affiliated to Zhejiang Shuren University Shulan International Medical College, Hangzhou 310022, China; 4 Jinan Microecological Biomedicine Shandong Laboratory, Jinan 250021, China

**Keywords:** *Lactobacillus reuteri*, acute lung injury, gut microbiota, inflammation

## Abstract

Acute lung injury (ALI) causes lung inflammation and edema as well as resulting in gut microbiota disorder. Probiotics, however, can improve the gut microbiota composition and modulate its immune response, playing an important role in ALI pathogenesis. Therefore, our study aims to investigate the effect of *Lactobacillus reuteri* on Lipopolysaccharide (LPS)-induced ALI in mice and to probe the mechanism of its synergistic modulatory effect on the lungs and intestines. We assessed the therapeutic effects of *L. reuteri* in the ALI mouse model by histopathology, alveolar lavage fluid and serum inflammatory factor analysis and explored microbiome and transcriptome alterations. *L. reuteri* intervention effectively attenuated lung tissue injury and significantly reduced the LPS-induced inflammatory response and macrophage and neutrophil infiltration. Additionally, *L. reuteri* improved the intestinal barrier function and remodeled the disordered microbiota. In conclusion, our study showed that *L. reuteri* attenuated the inflammatory response, ameliorated the pulmonary edema, repaired the intestinal barrier, and remodeled the gut microbiota in ALI mice. This study provides new perspectives on the clinical treatment of ALI.

## 1. Introduction

Acute lung injury (ALI) is an acute respiratory failure due to diffuse interstitial lung lesions caused by damage to the pulmonary vascular endothelium and alveolar epithelium by noncardiogenic factors such as severe infections, shock, trauma, and burns. The Berlin definition published in 2012 defines ALI as mild or moderate acute respiratory distress syndrome (ARDS) [1]. The main manifestations of ALI/ARDS include increased lung permeability, inflammatory cell infiltration and alveolar edema, which are highly susceptible to the development of critical illness [2]. In addition, the mortality rate of ALI/ARDS is approximately 40% in numerous studies [3,4]. Currently, ALI/ARDS treatment is based on mechanical ventilation and fluid management without effective clinical therapies, resulting in a poor prognosis for most patients [5].

In recent years, the interactions between lung disease and gut microbiota has been frequently reported in studies [6,7,8]. One study showed that an increased permeability of the intestinal wall in patients with ARDS and enrichment of intestinal bacteria in the lung microbiota is closely related to the severity of acute systemic inflammation [6]. Another study indicated that the gut microbiota composition was closely associated with inflammatory factor levels and inflammatory markers in COVID-19 patients, suggesting that there may be a link between the gut microbiota and host immune response [9]. These studies are evidence that the gut microbiota may be important in the treatment of ALI. Recently, numerous studies have noted the potential of probiotic supplementation in the clinical treatment of patients with ALI/ARDS, which can improve patient symptoms, enhance immunomodulation, and remodel the gut microbiota [10,11,12,13]. However, only a few probiotics have been proved to be protective in patients with ALI.

*Lactobacillus* is widely used as an important probiotic that helps protect the intestinal mucosal barrier, improve the host’s immune function, and maintain the intestinal microbial balance [14,15,16,17]. *Lactobacillus reuteri* as an important member of *Lactobacillus*, has been shown to maintain intestinal epithelial homeostasis and repair pathological damage to the intestinal mucosa [14]. In addition, in another study, *L. reuteri* was revealed to inhibit the production of inflammatory cytokines, increasing the survival rate of ADRS mice [11]. Moreover, Milagros Griet et al. reported that *L. reuteri* CRL1098 soluble factors reduced inflammatory factors released by LPS-stimulated mouse macrophages [18]. In conclusion, *L. reuteri* provides new approaches for ALI treatment.

In our study, we intervened with *L. reuteri* in LPS-induced ALI model mice with the aim of exploring the effects of *L. reuteri* on mice with ALI and clarifying the possible mechanisms involved through multiomic analyses, including 16S rRNA sequencing, transcriptomics, and metabolomics.

## 2. Materials and Methods

### 2.1. Strain and Culture Conditions

Following previous research, the probiotic strain *L. reuteri* was obtained from Protectis baby drops (Biogaia AB, Stockholm, Sweden) [19]. The strain was incubated anaerobically in a Man–Rogosa–Sharpe (MRS) broth at 37 °C for 24 h. Afterward, the medium was centrifuged at 4000× *g* for 10 min, and then washed and resuspended in phosphate buffered saline (PBS) for gavage.

### 2.2. Animal Experiments

**Figure 1:**
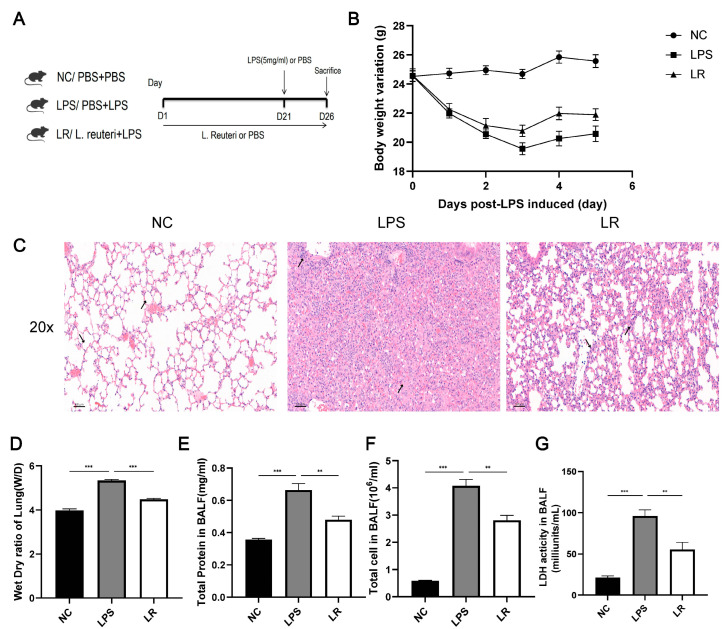
*L. reuteri* attenuates lung injury in ALI mice. Experimental flowchart (**A**); Changes in body weight (**B**); (**C**) Images of HE staining of lung tissue (magnification × 20, scale = 50 μm); (**D**) lung dry/wet ratio in mice; Concentration of total protein (**F**), total cell count (**E**), and LDH activity (**G**) in BALF; Data are presented as mean ± SEM. ** *p* < 0.01, and *** *p* < 0.001. NC, PBS + PBS group; LPS, PBS + LPS group; LR, *L. reuteri* + LPS group.

The model was induced by intratracheal administration of LPS (Sigma–Aldrich, Gillingham, UK). C57BL/6 male mice (6 weeks, 22 ± 0.5 g) were housed in a specific pathogen-free room with a controlled 12-12 h light-dark cycle and a constant temperature of 22–24 °C. After acclimatization feeding, the mice were randomly divided (Figure 1A): the NC (PBS + PBS) group, the LPS (PBS + LPS) group, and the LR (*L. reuteri* + LPS) group (*n* = eight per group). The LR group was gavaged daily with *L. reuteri* suspension, whereas the NC and LPS groups were treated with PBS as a placebo treatment for 21 days. On day 22, mice in the LPS and LR groups were induced with 0.5 mL of 5 mg/kg LPS, and the NC group received 0.5 mL of PBS. After 5 days, mice were euthanized by injection of sodium pentobarbital, the left lungs were washed with PBS to collect bronchoalveolar lavage fluid (BALF), and samples of blood, lung, and intestinal tissues were collected. All animal experiments were reviewed by the Ethics Committee (No. 2023-969).

### 2.3. Histopathologic Evaluation

Lung and colon tissues were fixed in 10% formalin and after 24 h we dehydrated them through a graded alcohol series and embedded them in paraffin. We cut the tissue wax block into 3 μm slices and stained them with hematoxylin and eosin (H&E). We stained the lung tissue with anti-F4/80 and anti-myeloperoxidase (MPO) antibodies; five representative fields of view were randomly selected at 40× magnification and quantified by calculating the percentage of positively stained areas in the field of view by ImageJ, and the pathology scores were assessed by two independent professors of pathology in a double-blind manner. Colon tissue sections were fluorescently stained with anti-ZO-1, anti-occludin and anti-MUC2 antibodies to visualize the intestinal barrier. We scanned the images using a Pannoramic MIDI scanner (3DHISTECH, Budapest, Hungary).

### 2.4. Calculation of Lung Tissue W/D Ratio

Mouse right middle lobe lung tissue was collected and its wet weight (W) was measured immediately after suction drying of the surface water. The tissues were then dried at 70 °C for 72 h to measure the dry weight (D). Tissue edema was assessed by calculating the W/D value.

### 2.5. BALF Assay Analysis

The BALF was centrifuged at 800× *g* for 15 min, erythrocytes were lysed and resuspended in 1000 μL of PBS, and then the number of cells was measured using an automated cell counter (Thermo Fisher Scientific, Waltham MA, USA). Meanwhile, we used a BCA protein assay kit (P0011, Beyotime Biotechnology, Nantong, China) to test the total protein level in the supernatant. In addition, the levels of lactate dehydrogenase (LDH) activity, IL-1β and IL-6 in the BALF were measured using ELISA kits (Abcam, Cambridge, UK).

### 2.6. Hematological Assay

The LPS binding protein (LBP) concentration was measured using an ELISA kit (ab269542, Abcam, Cambridge, UK), and a mouse Cytokine 23-Plex Assay (Kit Bio-Rad, Hercules, CA, USA) was used to measure the concentration of serum inflammatory factors. All procedures were performed according to the kit manuals.

### 2.7. Real-Time Fluorescent Quantitative PCR Analysis

We used an RNeasy Plus Mini Kit (74134, Qiagen, Valencia, CA, USA) to extract RNA from colon tissues and converted it to cDNA (500 ng RNA per 10 μL of cDNA system), and then the mRNA levels were detected in the ViiA7 real-time PCR system (Applied Biosystems, Waltham, MA, USA). We used the mRNA expression level of Gapdh as the control target gene. The sequences of gene primers are shown in Supplementary Table S1.

### 2.8. Microbial Composition Analysis

We collected mouse colon contents and extracted DNA from them by using a DNeasy PowerSoil pro kit (47016, Qiagen, Hilden, Germany). PCR amplification was then performed using primers for the variable regions V3 to V4 of the 16S rRNA gene (Supplementary Table S1). After identification, libraries were constructed, and the PCR products were sequenced on an Illumina NovaSeq 6000 platform (Illumina Inc., San Diego, CA, USA).

The data were imported into QIME2 for processing, clustered into amplicon sequence variant (ASV) groups by using the DADA2 plug-in and categorized according to the Silva 138 database in QIME2. Finally, α-diversity and β-diversity were assessed by QIME2 software. Differences between taxa were also analyzed using linear discriminant analysis (LDA) effect size (LEfSe). Raw data were uploaded to the SRA database (PRJNA1015362).

### 2.9. Metabolomic Analysis

Liquid chromatography mass spectrometry (LC–MS) metabolic analysis was performed on the cecum contents. The samples preparation method was as before [20] and the results were subsequently analyzed in an ACQUITY UPLC I-Class liquid chromatograph mass spectrometer (Waters Corporation, Milford, MA, USA).

### 2.10. Transcriptome Analysis

Lung tissue RNA was extracted, and a library was constructed and sequenced as described previously [21]. Subsequently, between-group differential transcript analysis was performed using the R package DESeq2, genes for which DESeq2 determined a *p*-value < 0.05 were considered differentially expressed. We used the Kyoto Encyclopedia of Genes and Genomes (KEGG) pathways for enrichment analysis of differential genes. The data were uploaded to the SRA database (PRJNA1015376).

### 2.11. Statistical Analysis

We analysed the data using SPSS software (version 20.0; SPSS, Chicago, IL, USA). We tested the date normality by using the Kolmogorov Smirnov test, and then analyzed them using the Mann–Whitney U test or Student’s *t* test. The results were expressed using the mean ± standard error of the mean (SEM), and *p* < 0.05 was considered statistically significant. Images were prepared in a GraphPad Prism 9.0 and R (version 4.2).

## 3. Results

### 3.1. L. reuteri Attenuates Lung Injury in ALI Mice

We established the ALI model by intratracheal injection of LPS (5 mg/kg) (Figure 1A). According to Figure 1B, pregavage of the probiotic *L. reuteri* for 21 days had no effect on the body weight, whereas after LPS induction, the weight of the LPS group was significantly reduced, but the *L. reuteri* intervention alleviated this symptom.

To assess the condition of the lungs of the mice, we stained the lung pathology sections with H&E. The alveolar wall in the LPS group was thickened and associated with diffuse interstitial infiltration. In contrast, the alveolar wall was significantly thinner in the LR group and the immune cell infiltration was reduced (Figure 1C). In addition, we also collected the BALF to measure the total protein content (Figure 1E), total cell count (Figure 1F), and LDH activity (Figure 1G) to further assess lung injury. Compared to the NC group, the total cell count, total protein level and LDH activity were increased in the LPS group, while the LR group showed a decrease in all of these parameters. In addition, *L. reuteri* treatment attenuated the degree of pulmonary oedema, with an increase in the W/D value of the lung tissue in the LPS group and a decrease in the LR group. (Figure 1D).

Overall, *L. reuteri* attenuated LPS-induced ALI in mice, especially in terms of pulmonary edema and alveolar epithelial permeability.

### 3.2. L. reuteri Reduces the Inflammatory Response Caused by LPS

**Figure 2:**
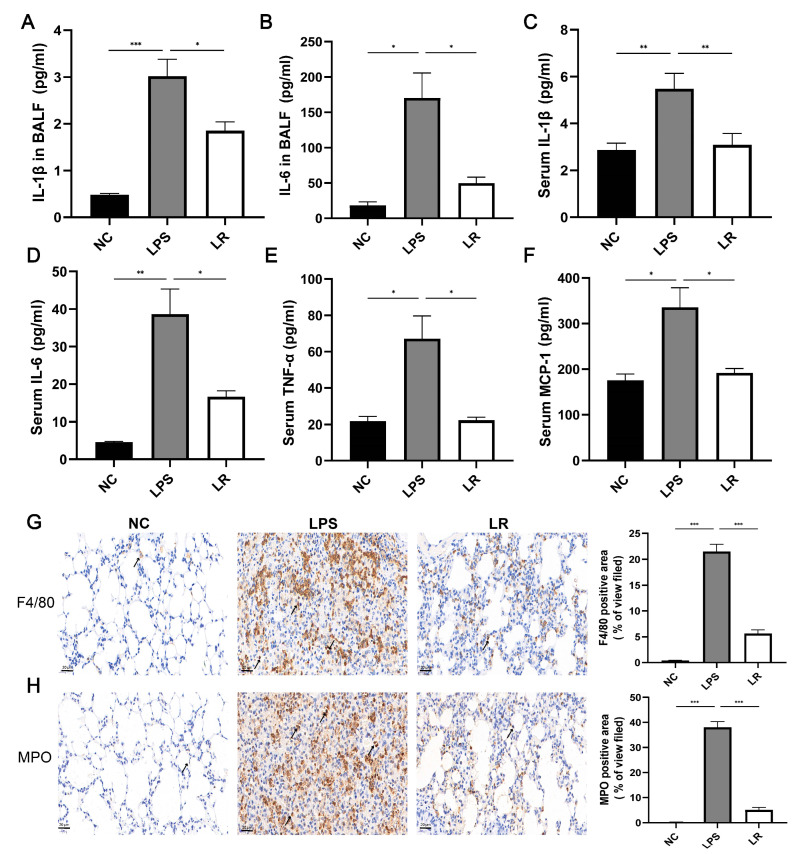
*L. reuteri* reduces the inflammatory response caused by LPS. IL-1β (**A**) and IL-6 (**B**) concentrations in BALF; serum levels of IL-1β (**C**) IL-6 (**D**), TNF-α (**E**), and MIP-1 (**F**). Representative images of F4/80 (**G**) and MPO (**H**) immunohistochemical staining (magnification × 40, scale = 20 μm); *n* = three per group and five randomly selected visual fields. Data are presented as mean ± SEM. * *p* < 0.05, ** *p* < 0.01, and *** *p* < 0.001. NC, PBS + PBS group; LPS, PBS + LPS group; LR, *L. reuteri* + LPS group.

Inflammatory cell infiltration is an important feature of the LPS-induced ALI model, and we assessed the protective effect of *L. reuteri* by measuring inflammatory markers in serum and alveolar lavage fluid. As shown in Figure 2C–F, we observed that serum levels of proinflammatory factors were elevated in the LPS group; however, these proinflammatory factors were significantly decreased after *L. reuteri* intervention. In addition, compared to the NC group, the levels of inflammatory factors in the BALF were elevated in the LPS group, whereas they were down-regulated in the LR group after the *L. reuteri* intervention (Figure 2A,B).

In addition, to further evaluate the effects of lung macrophages and neutrophils on the ALI model mice after LPS induction, we stained the lung tissues using anti-F4/80 and anti-MPO antibodies. The LPS group showed significant enhancement of macrophages and neutrophils, whereas the LR group showed reduced recruitment of macrophages and neutrophils (Figure 2G,H). In conclusion, *L. reuteri* attenuated LPS-induced pulmonary and systemic inflammation in mice.

### 3.3. L. reuteri Reduces Intestinal Damage and Repairs Intestinal Function

**Figure 3:**
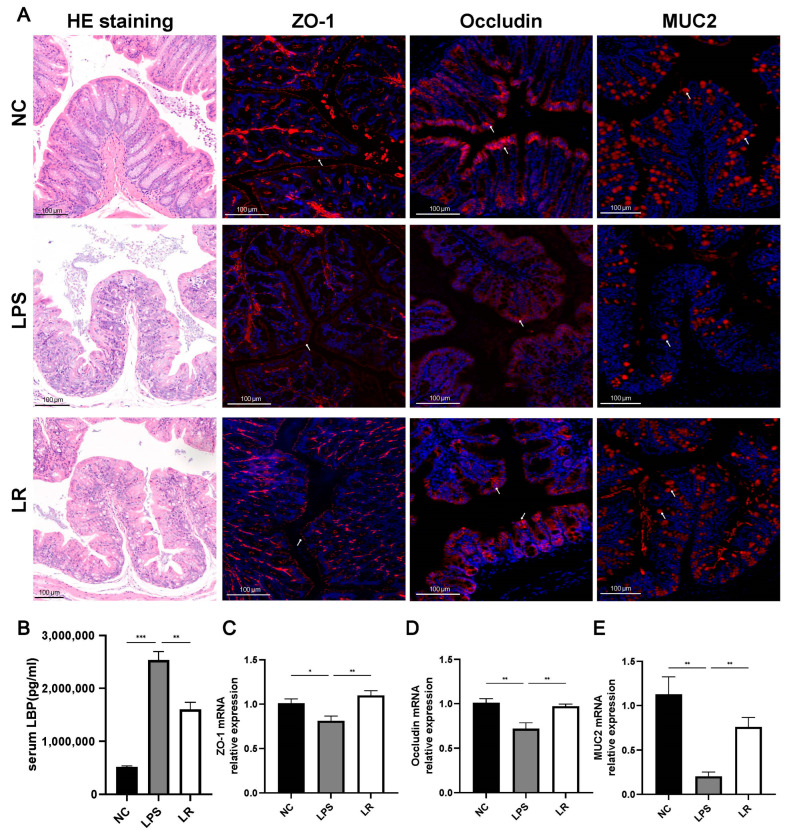
*L. reuteri* reduces intestinal damage and repairs intestinal function. (**A**) Representative images of HE staining (magnification × 10, scale = 100 μm) and immunofluorescence staining for ZO-1, occludin, and MUC2 of colon tissues (magnification × 15, scale = 100 μm). (**B**) Serum LBP levels were measured by ELISA. The mRNA levels of ZO-1 (**C**), occludin (**D**), and MUC2 (**E**) (red indicate positive areas). Data are presented as mean ± SEM. * *p* < 0.05, ** *p* < 0.01, and *** *p* < 0.001. NC, PBS + PBS group; LPS, PBS + LPS group; LR, *L. reuteri* + LPS group.

Systemic inflammation induced by lung injury disrupts the intestinal barrier integrity, which increases the permeability to intestinal microorganisms and their products and finally leads to exacerbation of the systemic inflammatory condition [6]. As shown in Figure 3A, colon HE staining indicated that villous atrophy and rupture, as well as epithelial barrier disruption, were clearly present in intestinal tissues of the LPS group, whereas *L. reuteri* markedly ameliorated the colonic epithelial damage. In addition, we further used immunofluorescence staining and q-PCR to assess the intestinal barrier function. The intestinal barrier was significantly impaired in the LPS group, with significantly lower mRNA expression levels of ZO-1 and occludin than those in the NC group, which was ameliorated by the *L. reuteri* intervention (Figure 3C,D). Meanwhile, the immunofluorescence results showed similar trends (Figure 3A).

Whereas disruption of the intestinal barrier leads to the translocation of bacteria and their metabolites into the bloodstream, thereby affecting systemic inflammation, CD14 is a receptor for the LPS and LBP complex [22]; therefore, we assessed bacterial translocation by measuring serum LBP concentrations. We observe that the serum LBP level in the LPS group was higher than in the NC and LR groups (Figure 3B). Meanwhile, we assessed intestinal permeability by detecting MUC2 levels in colonic tissues, and immunofluorescence staining in the LPS group showed significantly fewer positive areas (Figure 3A) and significantly lower levels of mRNA expression of MUC2 compared with that of the NC group, whereas the *L. reuteri* intervention markedly increased the level of MUC2 expression (Figure 3E).

In conclusion, LPS-induced intestinal mucosal damage in mice with ALI was severe; the intestinal barrier function was significantly disrupted, which may be closely related to the translocation of the intestinal flora, and *L. reuteri* was able to ameliorate the intestinal barrier dysfunction.

### 3.4. Effects of L. reuteri on the Composition of the Gut Microbiota

**Figure 4:**
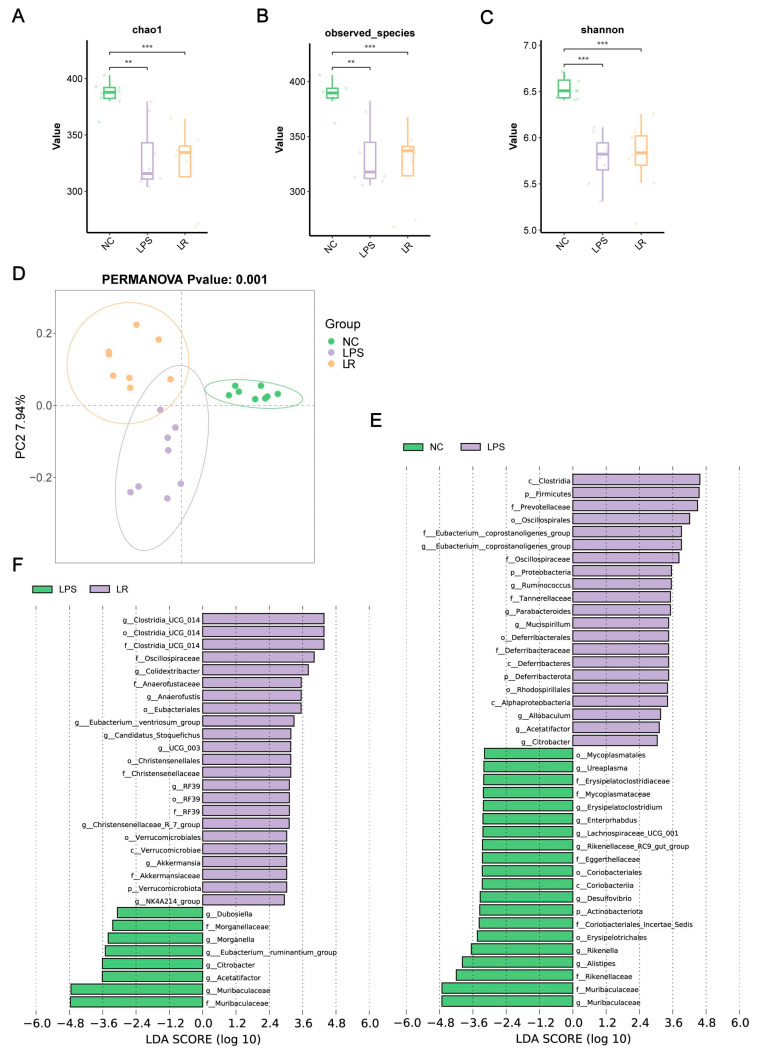
Effects of *L. reuteri* on the composition of the gut microbiota. Chao1 (**A**), observed species (**B**) and Shannon indices (**C**) of intestinal flora. (**D**) The PCoA plot based on binary Jaccard distances of three groups. LEfSe analyses plot between the NC and LPS groups (**E**) and the LPS and LR groups (**F**). ** *p* < 0.01, and *** *p* < 0.001. NC, PBS + PBS group; LPS, PBS + LPS group; LR, *L. reuteri* + LPS group.

To investigate the gut microbiota composition, we sequenced the 16S rRNA gene from mouse colon feces and obtained a total of 1,924,335 raw sequences from 24 samples. As shown in Figure 4A–C, our study demonstrated that the richness (Chao1 and observed species indexes) and diversity (Shannon index) of the gut microbiota were decreased in the LPS group; however, the diversity index was slightly increased after *L. reuteri* intervention. In addition, we further assessed the β-diversity of the microbiota by performing the PCoA on the samples based on binary Jaccard distances. As shown in Figure 4D, the microbiotas of the three groups were significantly separated (PERMANOVA, *p* = 0.001).

To further explore the key bacteria in gut microbiota, we used LEfSe analysis. Compared to the NC group, the phylum *Actinobacteriota*, class *Coriobacteriia*, and orders *Erysipelotrichales* and *Coriobacteriales* were depleted in the LPS group. Furthermore, at the family level, *Muribaculaceae*, *Rikenellaceae*, *Coriobacteriales_Incertae_Sedis*, *Eggerthellaceae*, *Mycoplasmataceae* and *Erysipelatoclostridiaceae* were also depleted in the LPS group. However, the phylum *Proteobacteria*, the phylum *Deferribacterota*, the class *Alphaproteobacteria*, the order *Rhodospirillales*, the family *Tannerellaceae*, the genus *Citrobacter* and the genus *Parabacteroides* were enriched in the LPS group (Figure 4E).

In addition, the phylum *Verrucomicrobiota*, the class *Verrucomicrobiae*, the order *Verrucomicrobiales*, and the family *Akkermansiaceae* were enriched in the LR group compared with those in the LPS group (Supplementary Figure S1). Additionally, the LR group was enriched for the genus *Clostridia_UCG_014*, the family *Oscillospiraceae*, the genus *Colidextribacter*, the family *Anaerofustaceae*, the order *Eubacteriales,* the genus *Eubacterium__ventriosum_group*, the genus *UCG_003*, and the family *Christensenellaceae.* Moreover, the LR group was depleted for the genus *Muribaculaceae*, the genus *Acetatifactoraceae* the genus *Acetatifactor*, the genus *Citrobacter*, the genus *Eubacterium__ruminantium_group,* the genus *Morganella*, and the genus *Dubosiella* (Figure 4F).

In conclusion, the richness and diversity of the gut microbiota in mice with ALI were disrupted, and *L. reuteri* intervention was able to reshape the gut microbiota.

### 3.5. Effects of L. reuteri on the Metabolic Composition in Mice with ALI

**Figure 5:**
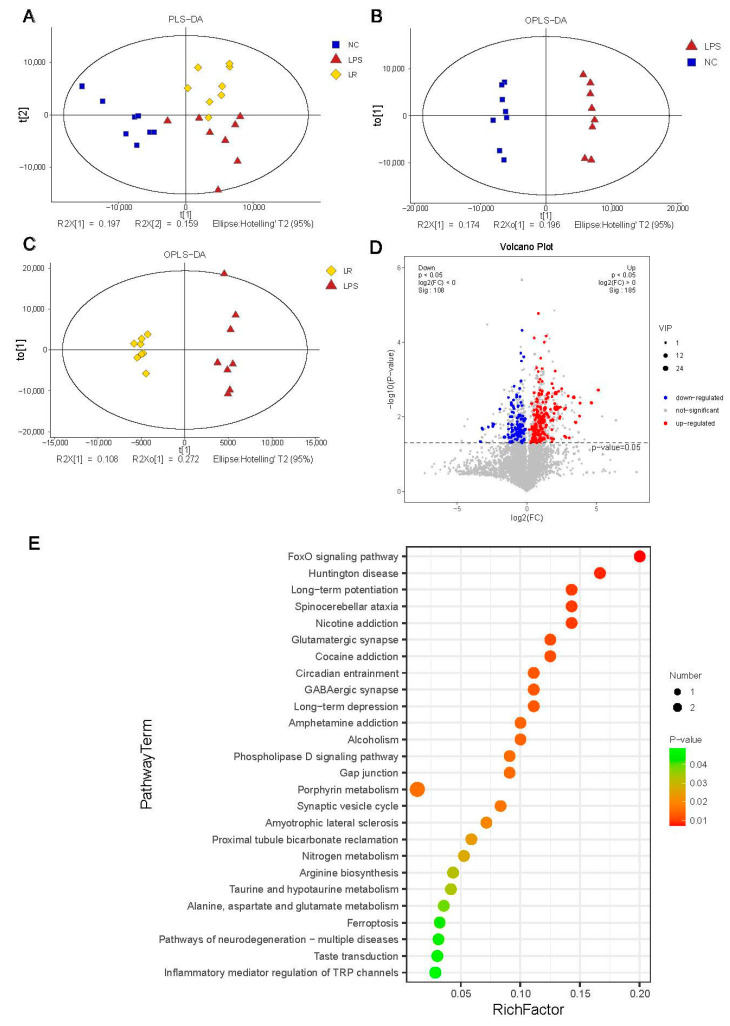
Effects of *L. reuteri* on the metabolic composition in mice with ALI. (**A**) PLS-DA plot of three groups. (**B**) OPLS-DA plot of the NC and LPS group. (**C**) OPLS-DA plot of the LPS and LR group. (**D**) Volcano plot of differential metabolites between the LPS and LR groups. (**E**) KEGG enrichment analysis of different metabolites between the LPS and LR groups. NC, PBS + PBS group; LPS, PBS + LPS group; LR, *L. reuteri* + LPS group.

To investigate the effect of *L. reuteri* on intestinal metabolites, we used LC–MS to analyze the contents of the mouse cecum. As shown in Figure 5A, metabolite level differences were observed in the partial least squares-discriminant analysis (PLS-DA) plot among the three groups, the NC, LPS and LR groups. The plot of orthogonal PLS-DA (OPLS-DA) between different groups also showed differences in metabolite levels between groups (Figure 5B,C). To further explore the differences in metabolite levels between the LPS and LR groups, we screened the results according to the thresholds variable importance in projection (VIP) >1, and *p* values < 0.05. Compared to those in the LPS group, a total of 185 metabolites was enriched and 108 metabolites were depleted in the LR group (Figure 5D).

In addition, we further examined the major metabolic pathways in the LPS and LR groups by KEGG pathway enrichment analysis, and identified a total of 26 downregulated metabolic pathways, including the FoxO signaling pathway, glutamatergic synapse, phospholipase D signaling pathway, ferroptosis, and inflammatory mediator regulation of TRP channels (Figure 5E). These pathways may be important for the amelioration of inflammation by *L. reuteri* in mice with ALI.

### 3.6. L. reuteri Regulates Lung Transcription in Mice with ALI

**Figure 6:**
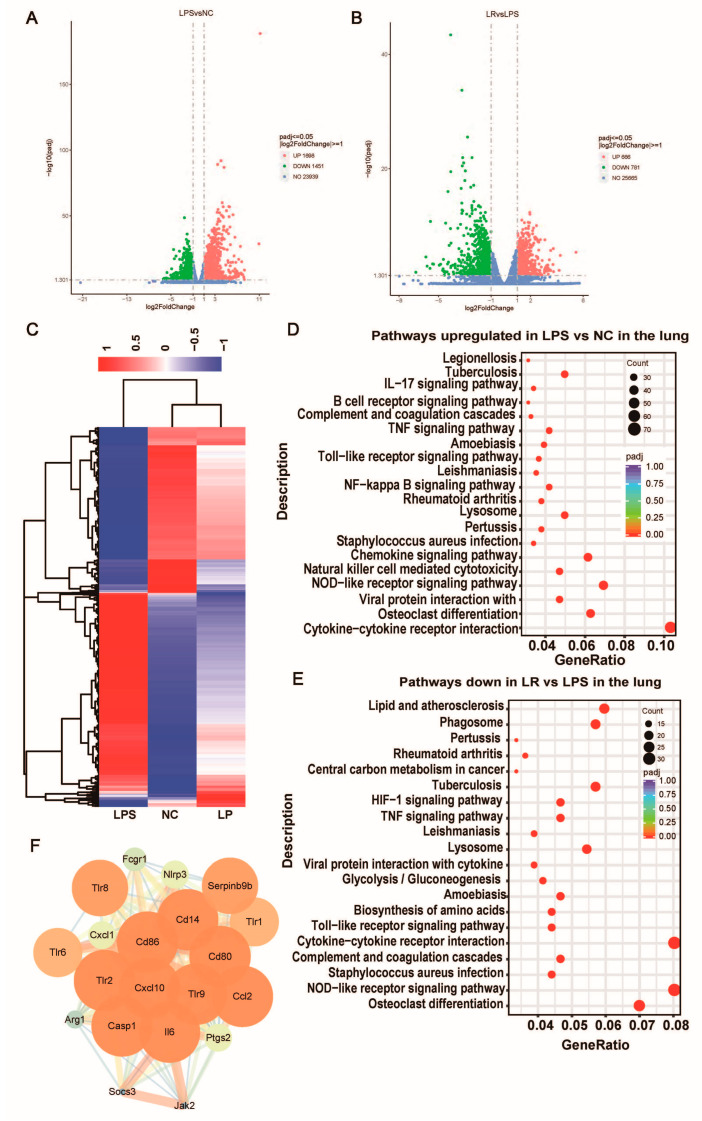
*L. reuteri* regulates lung transcription in mice with ALI. Volcano map of differentially expressed gene distribution between the NC group and LPS group (**A**) and the LPS group and LR group (**B**). (**C**) Heat cluster plot of the three groups. (**D**) Pathways in the lung were upregulated in the LPS group (compared with the NC group). (**E**) Pathways in the lung were downregulated by *L. reuteri* intervention (compared with the LPS group). (**F**) Visualization of the cluster network with the highest MCODE score. NC, PBS + PBS group; LPS, PBS + LPS group; LR, *L. reuteri* + LPS group. *n* = 3 in each group.

In an effort to comprehend the potential mechanism by which *L. reuteri* ameliorates LPS-induced lung injury, we conducted transcriptome profiling of lung tissue. Applying thresholds of |log2FC| ≥ 1 and *p*-value ≤ 0.05, we identified 27,088 differentially expressed genes (DEGs) between the LPS and NC groups. Specifically, there were 1698 upregulated and 1451 downregulated DEGs in the LPS group compared to the NC group. Furthermore, when comparing the treated with *L. reuteri* group to the LPS group, we saw that there were 666 upregulation and 781 downregulation DEGs (Figure 6A,B). Moreover, it was observed that the gene expression profile of the mice treated with *L. reuteri* exhibited a greater similarity to that of the mice in the NC group, as opposed to the untreated ALI model mice (Figure 6C). This finding suggests that *L. reuteri* has the ability to reverse the molecular changes associated with lung injury. Furthermore, we employed the KEGG pathway database to map the aforementioned genes, specifically emphasizing pathways that exhibited overlap. By applying an adj-*p* < 0.05 threshold, we identified a total of 42 downregulated inhibitory pathways, which possess meaningful implications. Notably, these pathways encompass the Toll-like receptor signaling pathway, cytokine-cytokine receptor interaction and NF-kappa B signaling pathway (Figure 6E). To further investigate the genes associated with protection, we further constructed a protein–protein interaction (PPI) network by categorizing the genes located within the downregulated pathways that displayed significant inhibition. Following the establishment of the PPI network, an examination of the cluster with the highest MCODE score was conducted to investigate the essential gene network. Furthermore, the identified key hub genes included Tlr8, Tlr1, Tlr9, Tlr6, Tlr2, Cd86, Cd14, Serpinb9b, Ccl2, Cxcl1, Cxcl10, Casp1, Fcgr1, Nlrp3, Socs3, Arg1, Ptgs2, and Jak2 (Figure 6E). Activation of the Toll-like receptors (TLRs) pathway triggers the release of proinflammatory cytokines, which may be important for the development of ALI.

## 4. Discussion

Numerous recent studies have mentioned that systemic inflammation caused by lung injury may lead to increased intestinal permeability and intestinal barrier damage, which increases intestinal permeability to the microbiota and its metabolites, resulting in the lungs accumulating intestinal bacteria, which can further contribute to lung disease exacerbation [23,24,25,26]. Crosstalk between the gut and the lungs is strongly correlated with lung disease severity, and the main direction of this crosstalk is from the gut to the lungs, although it cannot be ruled out that it occurs in the opposite direction [6,27,28,29]. Previous studies have shown that *L. reuteri* has significant potential for supporting intestinal epithelial regeneration, repairing the intestinal barrier, remodeling the intestinal microbiota, and inhibiting the production of proinflammatory factors, among many other activities [14,30,31]. However, the molecular mechanisms by which *L. reuteri* ameliorates ALI symptoms remain unclear. In our study, we evaluated the effect of *L. reuteri* intervention on ALI model mice, and elucidated the possible related mechanisms based on a multiomics approach (transcriptome, microbiome, and metabolome) (Supplementary Figure S2). The results indicated that *L. reuteri* ameliorated the symptoms of mice with LPS-induced ALI by suppressing lung inflammation, attenuating pulmonary edema, repairing the intestinal barrier, and remodeling the intestinal microbiota.

Inflammatory mediators are important in ALI/ARDS pathogenesis, especially proinflammatory factors, which can cause diffuse damage to alveolar epithelial and vascular endothelial cells, increase vascular permeability, and promote protein leakage, which further aggravates lung edema [32,33]. In our study, the HE staining showed that under the influence of LPS, the lung tissue structure of mice exhibited alveolar cell and interstitial damage, and *L. reuteri* intervention alleviated alveolar and interstitial edema and reduced inflammatory cell infiltration. In addition, we observed that *L. reuteri* reduced the levels of IL-1β and IL-6 in the lungs and found that *L. reuteri* intervention reduced capillary permeability by measuring BCA and LDH levels and total cell counts in the BALF. This finding is consistent with previously reported results that inhibition of inflammation is beneficial in reducing lung tissue damage [34]. Acute tissue injury or viral infection can activate immune cells, and IL-1β is mainly derived from activated macrophages [35,36], and can activate neutrophils, inducing a shock-like state in animal models [37]. In addition, elevated production of IL-6 has been suggested to be stimulated by IL-1β and TNF-α, and IL-6 can also be produced by a variety of cells, including macrophages and endothelial cells [38]; moreover, circulating levels of IL-6 have been considered by many studies to be a major predictive marker of ARDS severity due to different etiologies [39,40]. Therefore, we also performed immunohistochemical staining of lung tissues with anti-F4/80 and anti-MPO antibodies, which showed an increase in the amount of macrophages and neutrophils in the lung tissues of ALI mice; moreover, *L. reuteri* intervention reduced the levels of the above cells.

Our study also showed in lung transcriptome analysis that regulation of inflammation-related pathways may be the mechanism involved in attenuating lung injury by *L. reuteri* intervention. By analyzing differential genes through the KEGG pathway database, *L. reuteri* may attenuate LPS-induced lung injury by downregulating the Toll-like receptor and NF-kappa B signaling pathway. Previous research has shown that in mice with ALI, activation of the TLR signaling pathway in alveolar endothelial and epithelial cells phosphorylates NF-kB downstream of the pathway [41], which induces the release of inflammatory cytokines, leading to increased inflammation [42]. We further categorized genes within the downregulated pathways that were significantly inhibited in the LR group that may be involved in the alleviation of lung injury by L. reuteri. For example, the NLRP3 inflammatory vesicle induces caspase-1 activation and promotes IL-1β secretion, thereby affecting the inflammatory response [43]. Additionally, TLR2 is highly expressed in the lung macrophages and activated endothelial cells [44], and TLR2/Myd88/NF-κB activation (phosphorylation of the p65 subunit) induces secretion of MCP-1, TNF-α, IL-6, and IL-1β [45]. Our results for serum inflammatory factor levels were consistent with previous findings [45,46] and the *L. reuteri* intervention reduced these levels. Toll-like receptors are expressed on immune cells, which can activate the immune system, increase inflammatory response, disrupt the intestinal mucosal barrier and cause bacterial translocation [47,48]. Our results showed that *L. reuteri* intervention decreased serum LBP concentrations, increased the mRNA expression levels of intestinal ZO-1, occludin and MUC2, reduced intestinal permeability and repaired the intestinal barrier function.

Intestinal epithelial cells are arranged via tight junctions to form the first line of defense of the intestinal tract against infection, which forms a physical barrier, and both it and the microbial barrier are important intestinal barriers [49]. The microbial barrier is mainly composed of the intestinal microbiota, which can maintain the homeostasis of the intestinal mucosa and prevent the invasion of pathogenic bacteria, and the intestinal microbiota is relatively stable under normal conditions [50]. However, previous studies have shown that inflammation, immunity, and other factors may lead to disruption of and a decrease in the diversity of the intestinal flora, resulting in an increased susceptibility to harmful bacteria [51]. Consistent with previous findings, the 16S rRNA sequencing results showed a significant decrease in the abundance and diversity of the gut microbiome in ALI model mice. In addition, we observed that the LPS group was enriched in *Proteobacteria* at the phylum level, which has been shown in previous studies to produce endotoxin LPS and induce a strong inflammatory response favoring the invasion of harmful pathogens [52,53]. Moreover, the LPS group showed enrichment of the harmful bacteria *Parabacteroides* and *Citrobacter*. Previous studies showed that *Parabacteroides* was enriched in septic lung-injured rats [54], whereas *Citrobacter* was significantly enriched in mice coinfected with IAV–MRSA [55]. The LR group showed a decreased abundance of the harmful bacteria *Morganella* [56] and *Citrobacter* but an increased abundance of the beneficial bacteria *Akkermansia*, *Colidextribacter* and the *Eubacterium__ventriosum_group*. One study has shown that *Colidextribacte* reduces LPS-induced liver injury and inflammation by modulating hepatic TLR4 and NF-κB signaling [57]. *Akkermansia* has been suggested to ameliorate LPS-induced lung injury and inflammation in numerous studies [54,58]. Notably, the LR group, although not enriched for *L. reuteri*, showed an increased abundance of beneficial bacteria capable of ameliorating inflammation upon *L. reuteri* intervention.

Furthermore, *L. reuteri* intervention downregulated the expression of inflammatory mediators that regulate the TRP channel. Many studies have shown that TRP channels are expressed in various immune cells, including macrophages and T cells, and activation of TRPV1 in T cells may enhance inflammatory responses [59], but activation of TRPV4 leads to the release of the chemokine MCP-1, which may activate signaling pathways by inducing macrophage recruitment [60]. Thus, the gut microbiota is involved in various metabolic processes and these metabolites are important in the gut barrier function and the immune system [61,62].

## 5. Conclusions

In conclusion, our study explored the effects of *L. reuteri* on an LPS-induced ALI mouse model by means of multiomic analysis. The results showed that lung and systemic inflammation caused by LPS-induced ALI may result in a damaged gut barrier and a disturbed gut microbiota, while *L. reuteri* can repair the damaged gut barrier and remodel the microbiota, thereby reducing the inflammatory response. This study explores the potential mechanism by which *L. reuteri* reduces lung inflammation during LPS exposure, providing a theoretical basis for the ameliorative effects of probiotics on ALI symptoms and thus offering new perspectives on ALI/ARDS treatment in the clinic.

## Data Availability

Not applicable.

## Supplementary Materials

The following supporting information can be downloaded at: https://www.mdpi.com/article/10.3390/nu15194256/s1, Figure S1: (A) The differential species branching diagram between the NC group and the LPS group. (B) The differential species branching diagram between the LPS group and the LR group. Figure S2: Schematic diagram of *L. reuteri* intervention in LPS-induced acute lung injury. Table S1: Primer sequences used for real-time PCR analysis and 16S rRNA. Table S2: List of antibodies we used. Table S3: List of hub genes in PPI network of LR vs. LPS.
