# Supplementary material for: Lactobacillus reuteri Ameliorates Lipopolysaccharide-Induced Acute Lung Injury by Modulating the Gut Microbiota in Mice
Source: Nutrients. 2023 Oct 4;15(19):4256. doi: 10.3390/nu15194256 (PMC10574429; doi:10.3390/nu15194256)
Supplement: Supplementary file 1 [file nutrients-15-04256-s001.zip › nutrients-2637884-supplementary.pdf]

Supplemental Material

Supplemental Table S1. Primer sequences used for Real-time PCR analysis and 16S rRNA.

Gene	Forward Sequence (5'-3')	Reverse Sequence (5'-3')
**Gapdh**	TGGTGAAGCAGGCATCTGAG	TGCTGTTGAAGTCGCAGGAG
**ZO-1**	ACCCGAAACTGATGCTGTGGATAG	AAATGGCCGGGCAGAACTTGTGTA
**Occludin**	GGAGGACTGGGTCAGGGAATA	CGTCGTCTAGTTCTGCCTGT
**MUC2**	GAAGCCAGATCCCGAAACCA	GAATCGGTAGACATCGCCGT

Feces 16S rRNA	Forward Sequence (5'-3')	Reverse Sequence (5'-3')
**16S rRNA V3V4**	TACGGRAGCAGAG	AAGGTATCTAATCCT

Supplemental Table S2. List of antibodies we used

Antibody name	Catalog number	Manufacturer
**Anti-Myeloperoxidase antibody**	ab208670	Abcam, Cambridge, UK
**F4/80 Rabbit mAb**	70076S	Cell Signaling Technology, MA, USA
**Anti-ZO1 tight junction protein antibody**	ab221547	Abcam
**Anti-Occludin antibody**	ab216327	Abcam
**Anti-MUC2 antibody**	ab272692	Abcam

Supplementary Table S3. List of hub genes in PPI network of LR vs LPS.

Hub gene in cluster 1	Log2(fold change) in transcriptome (LR vs LPS)	P-value	Regulation
Tlr2	-1.880947518	2.13E-07	downreglated
Fcgr1	-3.161637411	3.48E-26	downreglated
Nlrp3	-1.497451222	0.000100162	downreglated
Tlr8	-1.414421998	6.29E-07	downreglated
Serpinb9b	-2.818046113	1.05E-06	downreglated
Cd14	-1.523084304	6.03E-05	downreglated
Tlr1	-1.798941854	9.62E-15	downreglated
Cxcl1	-2.192021089	0.008018021	downreglated
Cd86	-1.859782242	1.16E-10	downreglated
Cd80	-1.279444294	7.65E-05	downreglated
Tlr6	-1.714156609	1.00E-06	downreglated
Cxcl10	-4.623647623	2.53E-06	downreglated
Tlr9	-1.431158494	1.30E-06	downreglated
Ccl2	-3.077495474	3.38E-11	downreglated
Casp1	-1.218101908	0.001177543	downreglated
Il6	-4.453759419	9.76E-14	downreglated
Ptgs2	-1.234270113	8.92E-05	downreglated
Arg1	-4.58002191	0.000931932	downreglated
Socs3	-1.43788372	9.18E-10	downreglated
Jak2	-1.051230292	2.07E-05	downreglated

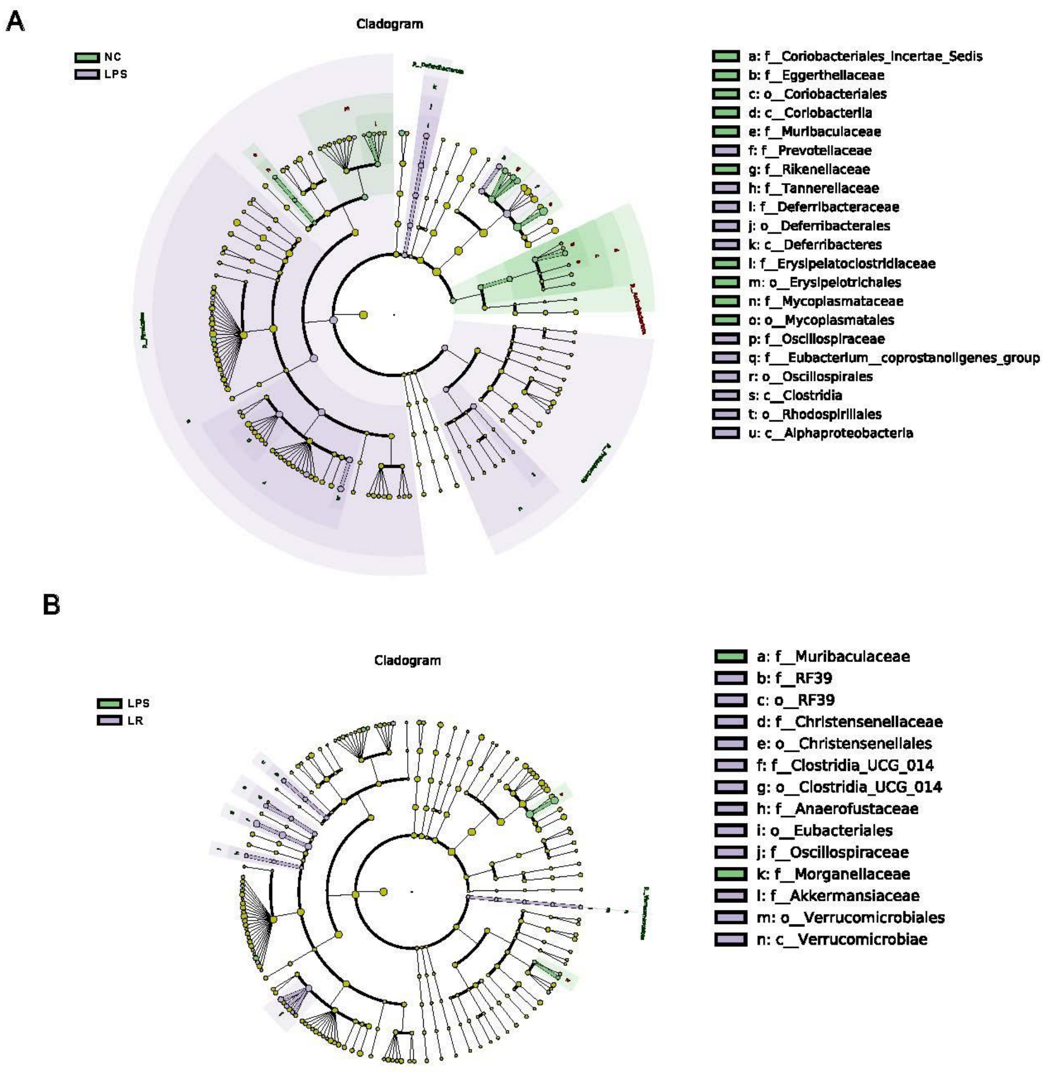

Supplementary Figure S1 (A) The differential species branching diagram between the NC group and the LPS group. (B) The differential species branching diagram between the LPS group and the LR group.

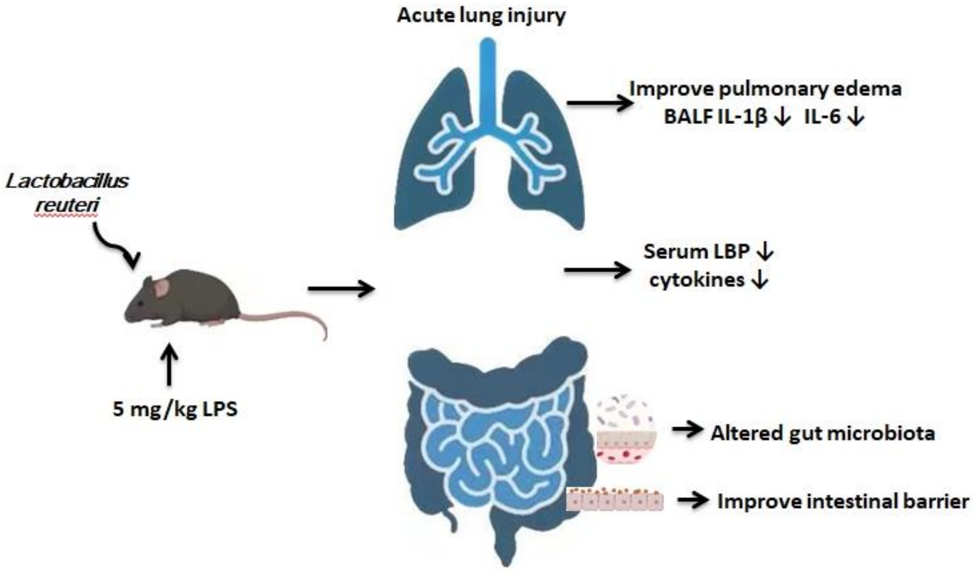

Supplementary Figure S2 Schematic diagram of *L. reuteri* intervention in LPS-induced acute lung injury.
